# Supplementary material for: Are carbon emissions trading and green financial instruments synergistic? -Comprehensive quantitative research based on content analysis
Source: PLoS One. 2024 Mar 7;19(3):e0298601. doi: 10.1371/journal.pone.0298601 (PMC10919601; doi:10.1371/journal.pone.0298601)
Supplement: S1 Data — (ZIP) [file pone.0298601.s001.zip › Data/地级市Z_F工作报告.zip!/╣π╢1⁄2/╣π╓▌╩╨/└┤╫╘╣1⁄2╓┌║┼í╛┬φ┐╦╩2╛▌═°í┐_2013.docx]

各位代表：

　　现在，我代表广州市人民政府，向大会作工作报告，请予审议，并请市政协委员和其他列席人员提出意见。

　　一、2012年工作回顾

　　2012年是新一届政府的开局之年，经济发展面临严峻挑战，全球经济持续低迷，国内经济下行压力增大，外贸出口和工业生产受到较大影响。同时，各项深化改革、改善民生、维护稳定的任务十分艰巨。面对复杂形势，我们认真践行科学发展观，贯彻市第十次党代会精神，以新型城市化发展为引领，统筹谋划发展战略，坚持为民，用心做事，锐意进取，攻坚克难，紧紧依靠全市人民，全力推动稳增长、促转型、惠民生、增后劲，较好地完成了市十四届人大一次会议确定的目标任务，各项工作取得新的成绩。

　　（一）经济保持稳定增长。我们坚持稳中求进的总基调，千方百计促进经济持续增长，质量和效益稳步提升。预计全市实现地区生产总值13500亿元，增长10．5%。地方财政一般预算收入1088亿元，增长11．1%。各项节能减排年度任务全面完成。主要采取了以下措施：加大投资和帮扶企业工作力度。落实市领导对口联系重点建设项目工作制度，多次召开市长办公会、现场会，协调推进项目建设。全市固定资产投资3758．39亿元，增长10．1%。举办“新广州·新商机”系列推介会和“百家知名企业羊城行”等招商活动，在穗投资的世界500强跨国企业增至232家。深入开展“暖企、暖区、暖项目”行动，协调解决企业用电、融资、用地等问题，增强经济发展活力。努力扩大消费需求。精心组织“广货全国行”、“广货网上行”、国际美食节、国际购物节等展贸活动，帮助企业开拓国内外市场。广州连续三年被评为福布斯中国大陆最佳商业城市。全市社会消费品零售总额5977．27亿元，增长15．2%；居民消费价格涨幅控制在3%左右。促进对外经贸与交流合作。精心组织企业参加各类经贸交流活动，大力拓展招商引资。预计全市实际使用外资45．7亿美元,增长7%。服务贸易发展和企业“走出去”步伐加快，商品出口总值约590亿美元，增长超过4%。加强和世界城市与地方政府联合组织（UCLG）的交流合作，创设“广州国际城市创新奖”，成功举办“一奖两会”，扩大了广州的国际影响力。努力营造经济发展良好环境。开展“三打两建”专项行动，成立市公安局食品药品犯罪侦查支队，实施建设社会信用体系、市场监管体系五年行动计划，进一步规范了市场秩序。开展安全生产“打非治违”和事故隐患排查治理。落实国家房地产宏观调控政策，房地产市场保持稳定发展。落实结构性减税政策，有力扶持中小微企业发展。深入实施质量强市战略，在全国专利执法绩效考核中我市位列副省级城市第一名。

　　（二）“三个重大突破”取得积极成效。战略性基础设施建设稳步推进。白云国际机场扩建工程、广州港出海航道三期、广州港南沙港区三期、贵广和南广铁路广州段等重点项目进展顺利，广珠铁路建成通车。地铁六、七、八、九号线和广佛线抓紧建设，新一轮地铁建设规划获国家批复。初步完成第二机场规划选址论证。增从高速公路全线通车，大广高速广州段项目开工建设。广州港货物和集装箱吞吐量持续增长，白云机场旅客吞吐量突破4800万人次。智慧广州基础设施建设加快推进，智慧广州战略与实践获巴塞罗那世界智慧城市大会奖。战略性主导产业不断壮大。发挥战略性主导产业发展资金的引导作用，推动产业加速向高端化、低碳化方向发展。成功举办首届中国（广州）国际金融交易·博览会，广州民间金融街首期、广州国际金融中心、广州股权交易中心、广州碳排放权交易所开业运营。总部企业集聚发展，会展业持续活跃，电子商务发展迅猛，唯品会、环球市场、欢聚时代3家电子商务企业在境外上市，黄埔云埔电子商务园区被授予“国家电子商务示范基地”。文化创意产业、旅游业保持兴旺。“广州价格”影响力进一步增强，中国木材（鱼珠）价格指数等成为行业风向标。预计全年服务业实现增加值8580亿元，增长11%，服务业增加值占地区生产总值的比重达63．6%。做大做强先进制造业，加快培育发展战略性新兴产业，东风日产花都第二工厂等项目建成投产，越秀区创意大道等8个项目完工，LGD8．5代液晶面板项目开工建设。8家企业成功上市，全市上市企业总数达91家。软件和信息服务业收入突破2100亿元，增长20%,广州获得“中国软件名城”称号。战略性发展平台建设成效明显。集中力量推进27个市级战略性发展平台建设。《广州南沙新区发展规划》获国务院批准，起步区建设前期工作同步展开，番禺区、南沙区行政区划调整顺利进行。出台《广州市中新广州知识城条例》、《中新广州知识城总体发展规划》，优质项目加快进驻。广州国际金融城、白云新城、白鹅潭商贸文化功能区、空港经济区、天河智慧城等重大平台建设顺利推进。科技创新能力进一步增强，广州荣获“国家知识产权示范城市”称号。广州超算中心、广州移动互联网（越秀）产业园等一批创新平台加快建设，国家“千人计划”南方创业服务中心正式挂牌，广州国际创新城等创新产业集群加快形成。

　　（三）城乡建设管理取得新进步。城乡规划和土地管理日趋完善。实施“一个都会区、两个新城区、三个副中心”城市功能布局规划，推进“三规合一”，新一轮城市总体规划编制取得阶段性成果。初步完成南沙新区、海珠生态城、广州国际金融城等示范区规划，完善了重点地区城市设计及控制性详细规划，修编生态城市、水系岸线、供电、供水等专项规划。新一轮土地利用总体规划获国务院批复。广州成为全国城乡统筹土地管理制度创新试点城市。成立市土地管理委员会，统筹土地资源开发利用，加强土地执法和耕地保护，顺利通过国家年度土地矿产卫片执法检查。出台《广州市农村村民住宅规划建设工作指引（试行）》，农村集体土地确权登记基本实现全覆盖，解决历史遗留房地产证办理工作基本完成，十区13361亩历史留用地98%已完成兑现或上报用地报批材料。万亩果园“只征不转”，开创生态用地保护新模式。建成数字广州地理空间框架，获得“全国数字城市示范市”称号。人居环境持续改善。实施空气污染综合防治10大行动。新增19个空气质量监测点，按新标准全面发布PM2．5等监测数据，全年环境空气质量优良率达98．4%。中山大道BRT试验线获联合国2012年应对气候变化环保“灯塔奖”。完成21个城市花景和158公里生态景观林带建设，推进岭南花园和十大森林公园建设。加大饮用水源工程建设，广州北江引水、万绿湖直饮水等工程取得进展。实施80公里江海堤防加固达标建设和13宗水库除险加固工程。推进东濠涌二期、荔枝湾涌三期等河涌综合整治，启动城市深隧系统排水工程规划建设，抓紧解决“水浸街”和污水处理问题。海珠湿地一期、西郊沙滩泳场建成开放，南沙滨海公共沙滩改造升级，云岭湖、金山湖全面完工。城市更新改造取得新进展。完善“三旧”改造政策，进一步强化了政府主导，突出公共利益。落实市人大1号议案决议，推进珠江黄金岸线改造与建设。成立同德围综合整治工作咨询监督委员会，同德围综合整治10项工程全面铺开。开展金沙洲地区综合整治，金沙洲大桥拓宽工程开工建设。交通综合整治系统推进。按照“建、增、管、限”思路，启动新一轮交通基础设施建设，新增70公里公交专用道，完成27个交通拥堵点治理。推进海珠区环岛新型有轨电车系统试验段规划建设。试行中小客车总量调控，新增停车泊位约4．5万个。破解“垃圾围城”取得进展。落实市人大2号议案决议，完善政策法规，广泛宣传发动，凝聚社会共识，推进垃圾分类处理，一批资源热力电厂建设项目按计划推进，餐厨垃圾处理设施和生活垃圾压缩中转站等建设改造项目全面铺开。城市精细化管理水平提升。开展城市管理网格化试点工作。持续整治“六乱”，设立流动商贩疏导区124处，安置疏导流动商贩近10万人。严查严控违法用地和违法建设力度加大。国家环保模范城市、国家卫生城市顺利通过国家复审。新农村建设取得成效。开展10个名镇、25个名村创建工作，完成了302个项目建设。发展都市型现代农业，新增农民专业合作社81家，实现“农超对接”、“农校对接”70多家。

　　（四）民生社会事业全面发展。去年市本级民生和各项公共事业投入385．1亿元，占市本级公共财政支出总额的75．2%。十件民生实事全部兑现。新建231家平价商店（专营区），扶持种养基地和冷藏设施建设项目98个，为16．7万低收入群体发放临时价格补贴2341万元。城镇企业退休人员月平均基本养老金水平提高到2614元；城镇平均低保标准从467元提高到530元，农村平均低保标准从377元提高到467元；低收入困难家庭认定标准从当地低保标准的1．2倍提高到1．5倍；“六类老人”政府购买居家养老服务标准提高100 元。市民基本公共卫生服务项目从9大项增至11大项，1000例贫困白内障患者复明手术全部完成，免费为10万户家庭提供出生缺陷干预服务。困难群众重特大疾病救助限额提高到每人每年14万元。普惠型学前教育、职业教育资助得到落实。筹集保障性住房46767套，新增解决 15798 户低收入家庭住房困难问题，廉租住房保障收入线标准从家庭人均可支配收入9600元以下提高到15600元以下，发放社会保险补贴 3．4亿元，帮扶20．22万失业人员再就业，城镇登记失业率为2．4%，广州被评为“全国创业先进城市”。对北部8个山区镇进行帮扶，被帮扶的贫困村和贫困户基本实现脱贫目标。开工建设87个公共服务基础设施，新开通50条公交线路。22个人行过街天桥加建雨篷项目全部完工。建成150个家庭综合服务中心，39个部门的571项事项接入市民网页，为55个社区装配自助金融终端设备。50个文化广场、288条健身路径、127个篮球场、9项乡镇农民体育健身工程顺利完工。建成绿道312公里，总里程达2174公里，居全省首位。城乡居民收入较快增长。城市居民人均可支配收入38108元，农村居民人均纯收入16898元，分别增长11．4%和14%。社会事业持续进步。巩固提高教育创强成果，越秀区被确定为省教育改革综合试验区，荔湾区、海珠区通过“省推进教育现代化先进区”督导验收，天河区被评为全国“两基”工作先进地区。改革完善学前教育体系，市财政投入学前教育超过3亿元。启动卓越中小学校长培养工程及新一轮“百千万人才培养工程”。中小学校舍安全工程三年规划全面完成，校车及校园安全工作得到加强。中等职业教育、高等教育、民办教育与特殊教育进一步发展，广州教育城建设规划启动。实施基本药物制度，加强基层医疗卫生机构建设，建成城市15分钟、农村30分钟的医疗服务圈。体育事业蓬勃发展，推进公共体育场馆免费和优惠开放，我市体育健儿在伦敦奥运会上取得两金一铜的优异成绩，广州职业足球卫冕中超冠军并夺取足协杯桂冠，职业羽毛球夺得全国超级联赛冠军，成功举办116项国际和全国体育比赛。国防动员工作得到加强，打击走私和海防工作取得新进步，双拥和优抚安置工作深入推进，第七次荣获全国“双拥模范城”称号。“羊城幸福家庭促进计划”扎实推进，优生优育水平不断提高。越秀区光塔街被评为“全国民族团结进步创建示范单位”。民族宗教、侨务、对台、应急、气象、地震、民防、统计、参事、文史、档案、修志、仲裁等工作得到加强，慈善、残疾人等事业全面发展。文化建设迈出新步伐。大力深化文明城市建设，我市在年度全国城市文明程度指数测评中成绩优良。“团一大”纪念广场、广州新图书馆、农民工博物馆建成开放，南越王宫博物馆等一批重点公共文化设施建设稳步推进。成功举办2012中国（广州）星海国际合唱锦标赛、第6届羊城国际粤剧节、第17届广州国际艺术博览会、2012中国（广州）国际纪录片节、第5届中国国际漫画节等重大文化活动。《广州大典》编纂和出版工作加快推进。对口帮扶和“双转移”工作成绩突出。圆满完成对口帮扶梅州、茂名、阳江扶贫开发“双到”三年工作任务，涌现出郭建南同志等一批先进典型。广州与梅州、阳江、湛江共建产业转移工业园取得新进展。对口支援新疆疏附工作受到自治区表彰，对口支援帮扶西藏波密、重庆巫山和广西百色工作成效显著。社会服务管理创新发展。开展幸福社区和美丽乡村创建试点工作。“一队三中心”全面挂牌。黄埔区综合化、网格化、信息化服务管理改革取得成效，南沙区、越秀区等社会服务管理创新试验稳步推进。全面实施社会组织直接登记，新登记数同比增长30%，社工专业人才和志愿者队伍进一步发展。

　　（五）政府自身建设进一步加强。各项改革稳步推进。全面开展新型城市化发展学习考察调研活动，制定系列政策文件，为改革创新注入强大动力。积极推进医药卫生体制改革、农村综合改革、自来水价格改革和电梯监管改革。全市停征行政性收费17项，为企业减负近2亿元。第五轮行政审批制度改革取得重大进展，行政审批和备案事项进一步压缩，网上办理范围逐步扩大。开展政府绩效管理和事业单位法人治理结构试点，推进事业单位分类改革，全市3306个单位完成了分类改革任务。法治政府、服务型政府建设成效明显。认真办理人大议案决议、代表建议、意见和政协提案，办复率达100%，办理质量和效率有新的提升。大力推进决策科学化、民主化、法制化，重大事项坚持集体决策，全年市政府召开全体会议4次、常务会议49次、市长办公会83次，完成地方性法规草案 5项，审议通过政府规章24 项和规范性文件 48项。实施市政府常务会议会后即时新闻发布和市政府领导定期新闻发布制度。组织开展全市性“大排查大评估大化解”活动，社会矛盾纠纷预防化解能力明显提高。法制宣传教育稳步推进，政务公开和纠风工作深入开展。预算执行情况审计进一步加强，市政府工作部门全部公开了部门决算和“三公”经费。廉洁广州建设扎实推进，成立市预防腐败局，建设反腐倡廉智能化管理平台，惩贪治腐力度明显加大。基层民主不断扩大，工会、共青团、妇联等人民团体的作用进一步发挥。

　　各位代表，过去的一年，我们经历了严峻考验，取得的成绩来之不易，这是党中央、国务院和省委、省政府及市委正确领导的结果，是全市人民团结奋斗和各方面支持帮助的结果。在此，我代表广州市人民政府，向全市人民、驻穗部队官兵、各民主党派、人民团体以及社会各界人士表示崇高的敬意！向关心和支持广州发展的港澳台同胞、海外侨胞、国际友人表示衷心的感谢！

　　在总结成绩的同时，也应清醒地看到，我市经济社会发展仍存在一些突出问题。有的重点项目推进不快，新引进的重大项目少，新的经济增长点不多，创新发展能力不强，财政增收压力加大，国资管理有待改进；生态环境有待改善，土地节约集约利用有待加强，交通拥堵、垃圾处理等问题亟待解决；城乡公共服务水平差距明显，统筹城乡发展任务艰巨；社会管理滞后，流动人员服务管理压力较大，安全生产形势不容乐观，民生福利水平与群众期待仍有一定距离。我们务必高度重视解决这些问题。

　　二、2013年工作安排

　　今年是贯彻落实党的十八大精神，全面推进新型城市化发展的关键一年。国内外经济形势依然错综复杂、充满变数，机遇与挑战并存。今年政府工作的总体要求是：围绕全面建设国家中心城市的目标和“率先转型升级、建设幸福广州”的核心任务，坚持为民，用心做事，科学把握“五位一体”总体布局，坚定不移稳增长、促转型、惠民生、增后劲，全面推进新型城市化发展，努力建设低碳、智慧、幸福的美丽广州，力争全市经济社会发展再上一个新台阶。

　　今年经济社会发展主要预期目标是：地区生产总值增长10%；公共财政预算收入按可比口径增长10%；固定资产投资增长13%；社会消费品零售总额增长14%；商品出口总值增长3%；万元生产总值能耗下降3．8%，化学需氧量、氨氮、二氧化硫和氮氧化物排放总量分别下降2%、4%、6%和5%；城市居民人均可支配收入增长11%，农村居民人均纯收入增长12%；城市居民消费价格指数涨幅控制在3．5%左右。全面实施新型城市化“1+15”政策文件，认真落实100项重点督办工作、10大产业项目、10项重点工程和10件民生实事。重点抓好八个方面工作：

　　（一）进一步增强经济发展动力

　　加快战略性基础设施建设。协调推进白云国际机场扩建工程、北部空铁联运国际交通枢纽中心和南航飞机维修基地建设，启动白云国际机场第四、五跑道建设前期工作。加快推进广州港深水航道拓宽工程和南沙港区三期工程。推进贵广、南广铁路广州枢纽工程、南沙港区疏港铁路建设。加快广州东部、北部、南部交通枢纽规划建设。做好穗莞深、广佛环线、广清城际轨道项目资金筹集和征地拆迁工作。加快国家批复同意的地铁线路可研报告报批进程。实现六号线一期开通运营，推进7条地铁工程建设。建设海珠区环岛路和环岛新型有轨电车系统试验段项目，建设黄埔疏港道路网络工程，推进打通一批断头路建设，加快洲头咀隧道、康王路下穿流花湖隧道、凤凰山隧道、新洲至化龙快速路、花城大道东延线、猎德大道北延线等工程的规划建设，开工建设新广从路快速化改造工程和增城沙庄至花都北兴公路二期工程。推进大广、广明高速公路建设。做好智慧城市基础设施建设，推进光纤到户、TD-LTE第四代移动通信网络、无线城市、云计算数据中心等重点工程。建设能源保障设施，天然气利用三期工程全面建成投产，电网建设加快推进。

　　推动产业转型升级。一手抓传统产业改造升级，一手抓战略性新兴产业培育布局。实施企业上市“双百”工程，推进“双转移”和第三批“退二进三”工作。制订优惠政策，优化总部经济发展环境，培育具有国际影响力的大型企业集团，壮大六大千亿元级产业集群。加快中心城区传统专业市场转型升级试点。促进信息化与工业化深度融合，重点加快自主品牌轿车研发和品牌建设，加大力度支持发展节能与新能源汽车，推进北汽集团华南生产基地整车项目首期工程、广汽丰田增加产品类型项目、广汽乘用车工厂产能扩建项目、东风日产纯电动汽车项目、黄埔新材料产业基地、白云民营科技园、南沙海洋工程装备制造基地等重大项目建设。开发利用海洋资源，大力发展海洋经济。

　　打造国际商贸中心。依托广州东、北、南的交通枢纽布局，规划建设会展副中心。加快广交会展馆四期扩建，建设广交会电子商务产业园和琶洲会展企业孵化基地。实施产业金融发展工程，力争新增小额贷款公司20家、股权投资机构30家。大力推进广州国际金融城建设。做好民间金融街二期建设，提升金融服务水平，打造民间融资“广州价格”。抓紧南沙现代金融服务区起步区建设，做优做强广州航运交易所，争取恢复设立广州期货交易所，引进一批股权投资机构和产业基金。完善广州金融创新服务区科技金融服务体系，加快广州股权交易中心发展。加大电子商务扶持力度，重点推进黄埔、荔湾国家级电子商务示范基地建设。推动天河路商圈、北京路广府文化商贸旅游区、广州南站商务区、番禺大道美食集聚区、老字号一条街、白云新城等一批商贸区建设，提升广州国际购物节、国际美食节、国际服装节、国际皮具节、国际灯光节等节庆活动的全球影响力。办好广州国际旅游展览会，提升旅游业发展水平。

　　深化国有企业改革发展。做实国有资产，加强资产统一监管，提高经营性国有资产收益。加强资本运作，加快推进国企改制上市，加快资本化、证券化步伐，完善法人治理结构，规范国企管理。鼓励优势国企“走出去”发展。

　　发展壮大民营经济。加强政策扶持，安排专项奖励资金支持民营企业发展。扩大民间投资，落实放开民资投资领域的实施细则和民营投资产业导向目录。积极构建多元化融资服务体系，加大土地利用计划统筹力度，努力解决民营企业融资难、用地难等问题。筛选一批行业骨干民企作为重点培育对象，扶持民营企业上市。完善中小企业社会化服务体系，优化民营企业发展环境。

　　扩大对外开放和区域合作。大力发展货物贸易和服务贸易，组织企业赴新兴市场国家和地区参展，争取新建1至2个海外商贸中心平台，努力扩大国际贸易规模。积极落实CEPA补充协议，把握南沙新区开发建设上升为国家战略的机遇，打造粤港澳全面合作示范区。继续推进广州马术场建设。以中新广州知识城为依托，提升国际经贸合作水平。继续加强与世界城市间的交流合作。加强与深圳的合作，落实广佛同城化、广佛肇经济圈建设合作框架协议。配合国家和省对我市实施《珠三角规划纲要》“四年大发展”考核验收工作，力争获得优异成绩。

　　推进战略性发展平台建设。把南沙新区开发建设作为广州1号工程。抓紧完善南沙新区规划体系，启动明珠湾区起步区建设，推进南沙新区交通枢纽设施建设。加快中新广州知识城国际招商和项目筹建。建设海珠生态城，做好城市新中轴线南段地区启动区、安置区规划选址和土地收储工作。加快增城经济技术开发区省级汽车（零部件）及新能源汽车产业园、省级LED产业基地、电子商务及物联网产业园等平台建设。扎实推进六大现代服务业功能区、九大创新型产业发展区、九大功能性发展平台建设，启动第二批战略性新兴产业基地认定工作，打造一批战略性产业集群。

　　（二）全面建设创新型城市

　　加快创新服务平台建设。重点建设广州超算中心、华南新药创制中心、广州市检验检测高技术服务集聚区等重大创新平台。推进广州高新区各园区、广州国际创新城等科技园区规划建设，完善相关配套服务设施，加强国际科技交流合作，打造国际科技孵化基地。加快建设一批国家级重点实验室，优化提升一批国家级工程技术研究中心。

　　推动创新型产业发展。实施创新型企业培育工程，加快推进国家科技与金融结合试点，设立和发展创业投资基金。落实支持科技企业成长的相关政策，加强知识产权保护，激励发明创造。以智慧城市建设推动电子信息产业结构优化升级，大力发展电子商务、软件、云计算、物联网、互联网、数字家庭等新兴产业。围绕建设广州健康医疗中心、国际生物岛，实施一批生物和健康技术重大项目。加快新环保、新能源、新材料、新电子、新医药与高端制造等产业技术创新。组织核心关键技术攻关，加快建成以协同创新为引领的自主创新体系。

　　实施人才集聚工程。充分发挥“千人计划”南方创业服务中心的作用，实施“红棉计划”、创新创业领军人才“百人计划”和珠江科技新星培育计划，大力推进高技能人才精工工程。创新“3+N”主题办会模式，不断提升广州留交会人才集聚效应。建设特色专业学院、技师学院，培养企业发展急需人才。鼓励企业实施股权激励以及分红激励试点，推动高等院校、科研院所与企业之间人才双向交流，支持知识城院士专家创新创业园建设和校地协同创新联盟工作，加快南沙新区“粤港澳人才合作示范区”建设。

　　提升城市质量竞争力。开展“全国质量强市示范城市”创建工作，稳步提升产品质量、服务质量、工程质量和环境质量。进一步完善政府质量激励制度，深入实施商标战略、品牌战略和标准化战略，增强“广州质量”、“广州标准”的国际国内影响力。

　　（三）统筹城乡一体化发展

　　优化城乡空间布局。全面推进“三规合一”工作，加快城市总体规划的编制报批与实施。深化完善都会区功能规划，开展两个新城区、三个副中心的控制性详细规划以及地下空间专项规划的编制工作，完善三大区域性交通枢纽规划设计。编制全市1142个行政村村庄规划。继续推进名镇名村和中心镇建设。发展绿色建筑，传承岭南建筑文化传统。

　　推进土地节约集约利用。全面开展城乡统筹土地管理制度创新，推进功能片区土地利用总体规划编制、城市生态用地差别化管理、低丘缓坡用地开发等创新试点工作。全面落实节约集约用地的各项措施。加快建立全市统一、城乡一体的土地交易平台，规范土地出让工作，提升市场配置土地资源的能力。坚持“政府主导、规划先行、成片改造、配套优先、分类处理、节约集约”，稳步推进城市更新和“三旧”改造工作。积极推进征地制度改革，盘活农村低效、闲置用地，提高集体土地的节约集约利用水平。严肃查处违法用地和违法建设。继续推进解决从化、增城历史留用地问题。进一步规范我市农村村民住宅的规划建设。

　　促进城乡产业融合发展。严格保护耕地，实行主要农产品最低保有量制度。加快发展都市型现代农业，促进农业企业集群发展。完善农业龙头企业协会制度，扶持农业龙头企业上市。探索推动农村宅基地和土地承包经营权抵押贷款试点。选择具备条件的镇（村）建设农村金融服务示范镇（村），打造农村金融综合服务集聚区和金融服务站。

　　建立农民增收长效机制。实施促进农民增收共富行动计划，稳步提高农民收入水平。推进农村人口向城镇、农村劳动力向二、三产业转移，促进农民就近就地创业就业。

　　提升城乡基本公共服务均等化水平。深化“村改居”管理体制综合改革。加强城乡教师交流，实施农村中小学教师、校长定期培训制度。建立城乡就业服务“网格化”管理机制，健全市四级就业服务体系。逐步整合城镇居民医保制度和新农合制度，推动医疗卫生资源向社区和农村倾斜，缩小城乡医保差距。完善城乡公共文化设施网络，大力开展“文化下基层”活动，推动公共文化服务覆盖城乡基层和外来务工人员。

　　（四）推进生态城市建设

　　倾力打造花城绿城水城。保护自然山体水体，打造生态保护区。推进一批岭南花园、森林公园以及城市主要出入口门户景观、带状绿地、立体绿化等建设。继续推进森林进城、森林围城，建设生态景观长廊。推进海珠生态城、芳村花地生态区、长洲湿地公园建设。进一步完善城市防洪排水系统，试点建设东濠涌深隧排水系统工程。加大农村污水治理力度，保障农村饮用水安全。全面开展土壤环境调查，加强农业面源污染控制。推进荔枝湾涌三期、东濠涌二期、石井河截污工程建设和猎德涌升级改造，推进花都湖、增城挂绿湖、智慧城东湖、黄埔龙头湖和万绿湖直饮水、广州北江引水、北部自来水厂等工程建设，整体提升水环境质量。深入发掘水文化，推进水博苑建设，形成“海、江、涌、湖、园、林、城”的整体景观格局。

　　巩固提升环境空气质量。大力调整能源结构，增加天然气、电力等清洁能源的供应量，加大力度控制煤炭消费总量。深化工业企业二氧化硫、氮氧化物、烟尘治理，实施工业锅炉、窑炉清洁能源改造。推广使用粤IV标准车用柴油，对柴油车实施国IV标准。加大环境监察力度，强化餐饮业油烟综合整治、扬尘污染控制和重点行业挥发性有机物排放控制，防治港口船舶交通废物和废气污染。加快广州市环境监测与预警中心建设。

　　（五）认真办好十件民生实事

　　一是完善医疗卫生服务。建立统一的“12320”卫生公益热线。在全市医疗机构推广预约挂号系统。免费为10万名适龄儿童提供窝沟封闭等口腔保健服务。免费为我市户籍及在我市居住半年以上的市外户籍、符合生育政策的夫妇提供孕前优生健康检查。二是提高养老服务和医疗救助水平。新建40个日间托老机构（项目）。动工兴建1万张养老床位。将农村“五保”供养标准提高到当地农村人均纯收入的70%。投入1500万元为约20万名困难群众购买重大疾病商业医疗保险。三是提高社会保险待遇。城乡居民社会养老保险基础养老金提高15%以上。四是推进城乡居民住房建设。新建和筹集保障性住房1．6万套，向1500户困难家庭发放住房租赁补贴，完成2012年在册城市零散危破房9．88万平方米改造任务。完成1万户农村泥砖危房改造工程。继续开展以北部山区为重点的农村扶贫开发工作。五是促进就业增长。投入4．6亿元就业专项资金，帮助18万名城镇登记失业人员实现再就业。开发2万个公益性公共服务岗位，举办300场次就业专场招聘会，发放8万张职业技能培训券。实现全年城镇新增就业人数24万以上。城镇登记失业率控制在3．5%以下。六是加强“菜篮子”和食品安全建设。提高“菜篮子”产品自给率，创建3个蔬菜生产标准园和3个健康水产养殖示范场，规划建设一批大型现代化养猪场。启动市食品生产企业检测监测数据分析平台建设。新增市级餐饮服务食品安全示范单位270家、示范街区（美食广场）12条。七是促进公共交通发展。新增投放500台公交车，新增公交线路20条、水上巴士航线3条，新建和改造水上码头5个，新增新建公交专用道50公里。八是完善群众文体设施。大力开展文化惠民活动，安排群众文化活动1000场、专业演出20场、农村数字电影放映1．3万场、展览150场。进一步加大市属公共体育场馆亚运赛后惠民开放力度，享受优惠进场人数较2012年增加10%。新建100条健身路径、50片篮球场、50个社区体育活动室、26个幸福社区体育设施、9项乡镇农民体育健身工程。新增300公里绿道。九是推进垃圾分类处理。加快分类处理设施建设，建成第一资源热力电厂二分厂（李坑二厂）、基本建成大田山餐厨废弃物处理厂项目，开工建设市第四资源热力电厂项目。推进4个餐厨垃圾处理设施建设，改造升级垃圾压缩站40座，增加生活垃圾分类运输车200辆。十是推进社区金融服务。在全市100个社区设立社区金融服务点，统一社区金融服务标识，布设金融自助终端设备，全面提升社区金融服务水平。

　　（六）大力加强社会建设

　　完善公共教育体系。加快建设“省推进教育现代化先进市”。着力促进学前教育公益性普惠性发展，加强中小学教育均衡特色发展。发展老年教育，完善终身教育体系。推动中心城区优质教育资源向周边辐射，提升两个新城区、三个副中心的教育质量。加快建设广州教育城，打造职业教育基地。统筹发展特殊教育，促进民办教育健康协调发展。推进广州教育国际合作综合改革试验区建设。实施广州大学城提升计划，完善公建配套设施。

　　提高医疗卫生服务水平。继续深化医药卫生体制改革，完善基层医疗机构综合改革，建立健全全科医生制度。加快市、区（县级市）医疗设施建设，开展增城市、从化市县级公立医院改革试点。大力实施健康城市行动计划，加强职业病防治，倡导阳光健康生活方式。推进中医药强市和卫生信息化建设。

　　完善城乡社会保障体系。构建覆盖城乡全体居民、全民共享普惠的多层次社会保险保障体系。完善社会救助体系建设，扩大分类救助范围，将贫困家庭未成年人、残疾人和困难家庭等群体纳入分类救济范围。推进公益法律服务体系建设，进一步扩大法律援助覆盖面。加强残疾人社会保障和服务，加快广州爱心公园等残疾人基础设施建设步伐，推进无障碍设施进家庭。

　　深入开展平安广州建设。完善管理体制，加强流动人员与出租屋服务管理。深入推进严打整治，完善立体化社会治安防控体系，落实社会矛盾纠纷“大调解”机制，进一步增强人民群众安全感。推进“两建”工作，强化行政执法与刑事司法的衔接，严厉打击食品药品等违法犯罪行为，打造“食得放心”城市。加强交通安全宣传教育，提高市民交通安全意识。严厉整顿市场经济秩序，维护经济安全。强化公共安全体系和企业安全生产基础建设，遏制重特大安全事故。

　　打造幸福社区和美丽乡村。完成26个幸福社区和14个美丽乡村试点建设，启动300个幸福社区和122个美丽乡村创建工作。创新社会服务管理体系建设，落实政府购买社会服务制度。完善街道“一队三中心”体制，将家庭综合服务中心覆盖到每个镇。加强居委会规范化建设，优化社区治理结构，积极探索社区网格化、智慧化管理模式，健全新型社区服务管理体系。深入实施街道基础设施“五个一”工程，打造“一站式”社区服务管理综合信息平台。完善社区老年人福利服务设施，扶持发展社区居家养老，推进长者中心建设。

　　全面发展各项社会事业。推动全民健身深入开展，扶持职业体育和体育产业发展。办好广州国际马拉松、世界羽毛球锦标赛等重大体育赛事。继续做好人口计生工作。全面实施广州市妇女儿童发展规划。落实民族宗教政策，促进民族团结和宗教和谐。扎实开展“六五”普法工作。加强防灾减灾基础设施建设，提高“三防”工作水平。落实优抚安置政策，加强国防动员和“双拥”工作。做好海防、民防及打击走私工作。推进气象现代化。加大援疆工作力度，继续做好侨务、对台、统计、档案、保密、参事、文史、修志等工作。

　　（七）培育世界文化名城

　　提升市民文明素质。加强社会主义核心价值体系建设，大力弘扬“厚于德、诚于信、敏于行”新时期广东精神和“敢想会干为人民，和谐包容共分享”的广州亚运精神。深化文明城市建设，积极推进道德领域专项教育和治理，实施市民素质提升工程，建设志愿者之城，提高城市文明程度和社会道德水平。

　　打造新岭南文化中心。加快推进海上丝绸之路史迹“申遗”工作，以南海神庙、黄埔古港古村、沙湾古镇、西关广场等载体为依托构建大型主题文化区。建设十三行商埠文化街区，再现“十三行”历史风貌。建设广东省非物质文化遗产展示馆暨大小马站书院街和北京路广府文化核心区。擦亮“迎春花市”品牌。抓紧推进南汉二陵博物馆、粤剧艺术博物馆、广州华侨博物馆、广州海事博物馆、广州民俗博物馆、海云寺、南粤先贤馆、潘鹤雕塑艺术园、三元里抗英纪念馆等一批文化设施建设。推进“一园四馆”规划选址。

　　传承历史文化遗产。加强古城历史文化的保护利用。深入挖掘重点革命史迹的文化内涵。加大对粤剧粤曲、广东音乐、岭南美术等传统艺术及“三雕一彩一绣”等非物质文化遗产的保护利用力度。完成《广州大典》编纂工作，修编《广州文化遗产汇编》。

　　大力发展文化产业。理顺文化产业管理体制，完善文化产业发展政策措施。实施文化发展品牌战略，加强版权保护工作，创建全国版权示范城市。支持文化企业做大做强，鼓励文化企业兼并重组和上市融资。办好第九届中国音乐金钟奖等重大文化活动。加强文艺精品创作和文化名人培育，打造广州文化品牌。

　　（八）加强政府自身建设

　　深化行政管理体制改革。落实国务院批准广东省在行政审批制度改革方面先行先试的要求，继续深化行政审批制度改革，优化行政审批流程，推进政府绩效管理。建立完善统一规范的公共资源交易市场。上半年完成各区（县级市）大部门制改革。积极稳妥做好事业单位分类改革，继续推进事业单位法人治理结构试点和法定机构试点工作任务。扎实推进财税体制、投资管理体制、营业税改征增值税、文化和卫生体制等领域的改革。推进商事登记制度改革，建设社会信用体系，打造与国际接轨的营商环境。创新行政执法体制，开展荔湾区综合行政执法体制改革试点。积极推进南沙新区社会管理创新综合改革试点。

　　推进法治政府建设。坚持依法行政，认真执行市人大及其常委会的决议、决定，切实做好人大代表建议、意见及政协提案办理工作，定期通报办理情况。开展法治政府示范区、依法行政示范点创建工作，推进综合执法、市场监管领域管理体制改革工作。认真实施《广州市依法行政考核办法》。

　　提高决策科学化、民主化水平。完善政府重大行政决策咨询论证和公众征询制度，坚持问政于民、问需于民、问计于民、问效于民。深入推进“同心工程”，拓展民主党派和无党派人士参政议政渠道，充分发挥社会组织的作用，积极探索新的社会阶层人士有序政治参与的途径。努力畅通依法有序的民意表达渠道，积极推动网络问政，确保市民合法诉求、合法权益得到充分体现。

　　加强勤政廉政建设。切实改进工作作风，大力精简会议和文件。坚持艰苦奋斗、勤俭节约，坚决克服官僚主义和形式主义，狠抓工作落实。深化政务公开，完善政府信息发布制度，推进“阳光行政”，有效提升政府善治能力，以诚信政府建设带动诚信社会建设。加大各领域防治腐败力度，完善具有广州特色的惩治和预防腐败体系。深入推进纠风和专项治理，重点解决群众反映强烈的突出问题，提高政府公信力。完善各项民生事务的“一站式”政务服务中心，实行开放式办公、一站式服务、并联式审批，提高政务透明度和工作效率，建设廉洁高效的服务型政府。

　　各位代表，在新的起点上实现广州的新发展，是时代和全市人民赋予我们的光荣使命。展望未来，我们充满信心。让我们在市委的正确领导下，实事求是，励精图治，为推动广州经济社会发展再上新台阶，为广州人民的幸福安康而努力工作！
